# Sex-Based Differences in Autologous Cell Therapy Trials in Patients With Acute Myocardial Infarction: Subanalysis of the ACCRUE Database

**DOI:** 10.3389/fcvm.2021.664277

**Published:** 2021-05-26

**Authors:** Paul M. Haller, Mariann Gyöngyösi, Lourdes Chacon-Alberty, Camila Hochman-Mendez, Luiz C. Sampaio, Doris A. Taylor

**Affiliations:** ^1^Department of Internal Medicine II, Division of Cardiology, Medical University of Vienna, Vienna, Austria; ^2^Regenerative Medicine Research, Texas Heart Institute, Houston, TX, United States; ^3^Department of Advanced Cardiopulmonary Therapies and Transplantation, University of Texas (UT) Health Science Center, Houston, TX, United States

**Keywords:** cell based therapy, sex characteristics, cardiovascular diseases, clinical trials, acute myocardial infarction, sex differences, cell therapy

## Abstract

**Background:** Sex-based differences are under-studied in cardiovascular trials as women are commonly underrepresented in dual sex studies, even though major sex-based differences in epidemiology, pathophysiology, and outcomes of cardiovascular disease have been reported. We examined sex-based differences in patient characteristics, outcome, and BM-CD34+ frequency of the ACCRUE (Meta-Analysis of Cell-based CaRdiac studies) database involving patients with acute myocardial infarction (AMI) randomized to autologous cell-based or control treatment.

**Methods:** We compared baseline characteristics and 1-year follow-up clinical data: composite major adverse cardiac and cerebrovascular events (primary endpoint), and changes in left ventricular ejection fraction (LVEF), end-diastolic (EDV), and end-systolic volumes (ESV) (secondary efficacy endpoint) in women and men (*N* = 1,252; 81.4% men). Secondary safety endpoints included freedom from hard clinical endpoints.

**Results:** In cell-treated groups, women but not men had a lower frequency of stroke, AMI, and mortality than controls. The frequency of BM-CD34+ cells was significantly correlated with baseline EDV and ESV and negatively correlated with baseline LVEF in both sexes; a left shift in regression curve in women indicated a smaller EDV and ESV was associated with higher BM-CD34+ cells in women.

**Conclusions:** Sex differences were found in baseline cardiovascular risk factors and cardiac function and in outcome responses to cell therapy.

## Introduction

Cardiovascular disease (CVD), often thought of as a male disease, is the leading cause of death for women in the United States, killing more women than all forms of cancer combined (1, 2). For most CVDs, the epidemiology, pathophysiology, response to treatment, and outcomes differ for men and women (3). Women with acute myocardial ischemic syndromes tend to be almost a decade older than men with the same diagnosis, and they have higher rates of comorbidities including hypertension (3) and angina (4). Despite substantial improvements in cardiovascular death rates over the last decade, women have a worse prognosis after acute myocardial infarction (AMI) than men (5). These disparities may be attributed in part to sex differences with respect to the biological variance in gene expression and are manifested through various biochemical and cellular processes, including the myocardial adaption to disease (6).

Pharmacokinetic and pharmacodynamic responses can differ significantly between men and women (7). Although women have been historically underrepresented in cardiovascular trials (8), therapies often move toward standard of care for clinical use without accounting for these pharmacological differences (3, 9). When women receive treatment based on the data gained in clinical studies comprising primarily men, unanticipated events may occur related to sex-specific differences (10). Health risks were greater for women than for men in 8 of the 10 drugs withdrawn from the market by the Food and Drug Administration from January 1997 to December 2000 (11). Thus, sex differences have become a major consideration for improving patient care (12), and sex-related data should be captured early, starting in the treatment development phase and continuing throughout the clinical trial stage (13).

Regenerative medicine strategies are promising tools for repairing damaged tissues or organs, despite the conflicting results reported in clinical studies. Increased levels of circulating bone marrow (BM) progenitor cells reduce the risk of death from CVD in both sexes (14). Understanding the results from clinical studies and identifying factors that could affect clinical outcomes in cell-based therapies require further examination of published data. The question of whether sex-based differences in baseline characteristics and outcomes exist after cardiac regenerative therapy such as cell-based therapy has not been adequately studied (15). Therefore, in this study, we performed a *post-hoc* analysis of sex-based differences in the ACCRUE database involving patients with AMI who were randomized to either intracoronary cell therapy or control groups (16). We evaluated clinical data and the frequency of BM-CD34+ cells as an indicator of BM potency (17). This database includes 12 randomized, controlled, cell-based cardiac clinical trials comprising a large cohort of men and women with AMI and provides an excellent opportunity to examine sex-based differences.

## Methods

### Database

For this study, we extracted individual data on patient characteristics and outcomes from patients (*n* = 1,252) enrolled in 12 randomized controlled trials in the ACCRUE (Meta-Analysis of Cell-based Cardiac stUdiEs in Patients with Acute Myocardial Infarction Based on Individual Patient Data) database (16) (Table 1). The main data analyses and results have been published previously (16). Because the study showed no differences between cell therapy and control groups in any endpoint, we pooled data on the two groups and focused on sex-related aspects. The primary endpoint of the present analysis was sex-related major adverse cardiac and cerebrovascular events (MACCE; the composite of all-cause death, AMI, stroke, and target vessel revascularization). The secondary efficacy endpoints included changes in end-diastolic volume (EDV), end-systolic volume (ESV), and ejection fraction (EF) in women vs. men. The secondary safety endpoints were sex differences in freedom from death, combined hard clinical endpoints (death, AMI, or stroke), and target vessel revascularization (soft clinical endpoint).

**Table 1:** Number of women and men in clinical studies of cell-treated and control patients with acute myocardial infarction in the ACCRUE database.

**Clinical trial**	**Cell-treated**	**Control**	**Women in the study (%)**
	**(*n* = 767)**	**(*n* = 485)**	
	**Women/men**	**Women/men**	
Aalst Study	1/18	1/15	5.7
ASTAMI	8/42	8/42	16.0
BONAMI	10/42	5/44	14.9
BOOST	10/20	7/23	28.3
CADUCEUS	0/17	0/8	0.0
FINCELL	4/35	6/33	12.8
LATE TIME	12/46	3/26	17.2
REGENT	53/107	10/30	31.5
REPAIR AMI	18/83	19/84	18.1
SCAMI	3/26	5/8	19.0
SWISS AMI	24/109	11/56	17.5
TIME	10/69	5/36	12.5

Pre-specified subanalyses included the sex-associated number of BM-CD34+ cells and the correlation between BM-CD34+ cells and sex-related changes in cardiac functional parameters.

### Data Analysis and Statistics

The distribution of scale variables was assessed by visually inspecting histograms and quartile-quartile (Q-Q) plots. If it was determined that the data were normally distributed, a Student *t*-test was used to compare mean values between groups, after assessing their equality of variance with Levene's test. If the data were not normally distributed, then we used the Mann-Whitney test for comparisons. Continuous variables are presented as the mean ± standard deviation. Dichotomous variables were compared by Pearson's Chi-square test and are shown as frequencies.

Event rates over time for all four groups (cell-treated women, cell-treated men, control women, and control men) were plotted using the Kaplan-Meier method, and differences were assessed by the log-rank test. Analysis of co-variance models adjusted for sex and age were used to examine the effect of sex and other characteristics on scale variables.

A 2-sided *p*-value < 0.05 indicated statistical significance. Statistical analysis was performed with the use of R 3.4.3 (18).

## Results

### Baseline Characteristics

Of 1,252 patients enrolled in 12 trials of cardiovascular cell therapy with autologous bone marrow cells (*n* = 11) and autologous cardiospheres (*n* = 1), 1,019 were men (81.4%) and 233 (18.6%) were women. The percentage of women in the trials ranged from 0 to 31.5% (average, 16.1 ± 8.5%) (Table 1). As expected, the comparison of baseline characteristics showed that women were significantly older, had lower peak creatine kinase (CK) concentrations, and had a smaller left ventricular ESV and EDV than men (Table 2). Women had an increased prevalence of hypertension and had fewer diseased vessels. Men were more likely to have a history of smoking. No differences were observed between men and women in other comorbidities including diabetes and hyperlipidemia.

**Table 2:** Baseline characteristics of patients stratified by sex.

**Baseline characteristics**	**Women**	**Men**	***P*-value**
	**(*n* = 233)**	**(*n* = 1,019)**	
Treatment groups			0.126
Cell-treated, *n* (%)	153 (65.7)	614 (60.3)	
Control, *n* (%)	80 (34.3)	405 (39.7)	
Age (years)^*^	60.4 ± 10.7	56.5 ± 10.3	<0.001
Hypertension (%)	131 (57.2)	497 (48.8)	0.022
Hyperlipidemia (%)	116 (54.5)	499 (53.5)	0.796
Smoking (%)	100 (48.8)	539 (58.5)	0.011
Diabetes (%)	34 (14.8)	156 (15.3)	0.856
Number of diseased vessels (%)			0.004
1, *n* (%)	194 (85.1)	763 (76.1)	
2, *n* (%)	19 (8.3)	170 (17.0)	
3, *n* (%)	15 (6.6)	69 (6.9)	
Maximum CK (U/L)^*^	2524 ± 2083	3233 ± 2411	<0.001
Vessel target LAD (%)	137 (89.5)	549 (86.2)	0.270
Pre-EDV (mL)^*^	117.9 ± 38.8	148.9 ± 49.5	<0.001
Pre-ESV (mL)^*^	65.2 ± 29.9	84.8 ± 38.9	<0.001
Pre-EF (%)^*^	45.6 ± 12.1	44.2 ± 11.9	0.119
% CD34+ (*n* = 493)^*^	1.4 ± 1.3	1.4 ± 1.2	0.767
CD34+CD133+ (*n* = 206)^*^	1.2 ± 0.8	1.1 ± 0.6	0.434

* *Mean ± SD. CK, creatine kinase; EDV, end-diastolic volume; EF, ejection fraction; ESV, end-systolic volume*.

When comparing baseline characteristics of women and men in the cell-treated and control groups (Supplementary Tables 1, 2), we found that women were older than men in both cell-treated and control groups. CK, EDV, and ESV were significantly lower in women in both randomized groups. Women were more often hypertensive, had fewer diseased coronary vessels, and were less likely to be smokers than men only in the cell-treated group (Supplementary Table 1).

### Clinical Outcome Variables

**Figure 1:**
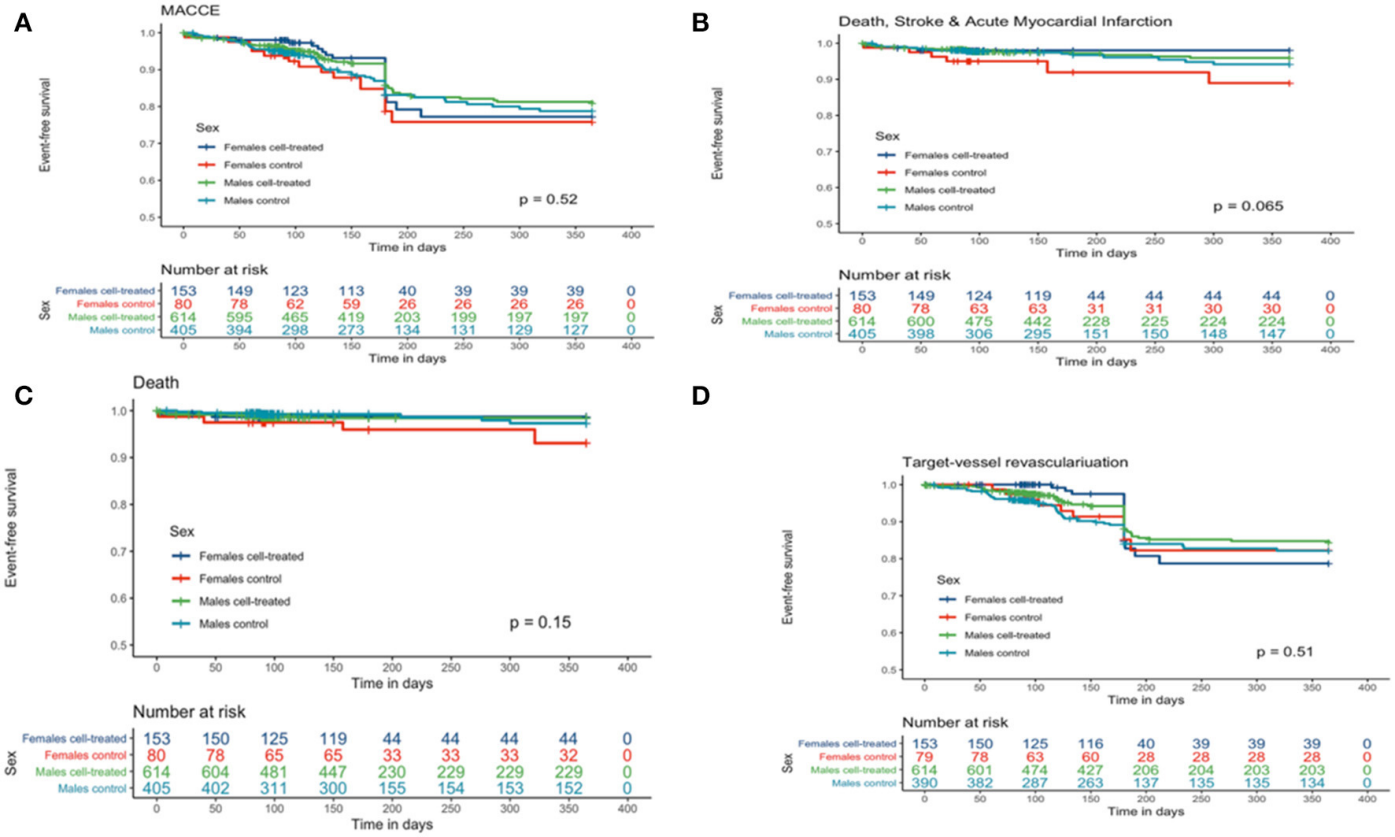
Kaplan-Meier curves for clinical outcomes of women and men in cardiac regenerative studies after acute myocardial infarction. **(A)** Major adverse cardiac and cerebrovascular event(s) (MACCE), including all-cause death. **(B)** Combined hard clinical endpoint (all-cause death, stroke, and acute myocardial infarction). **(C)** All-cause death. **(D)** Target vessel revascularization.

We found no significant differences in the time-dependent rates of in-hospital complications, MACCE, death, the combined hard clinical endpoint (death/stroke/AMI), and target vessel revascularization (Table 3 and Figure 1) at 1 year between men and women.

**Table 3:** Outcome characteristics in cell-treated and control groups stratified by sex.

	**Cell therapy**		**Control**		***P*-value**
	**(*n* = 767)**		**(*n* = 485)**		
**Follow-up clinical endpoints**	**Women**	**Men**	**Women**	**Men**	
	**(*n* = 153)**	**(*n* = 614)**	**(*n* = 80)**	**(*n* = 405)**	
In-hospital complications (%)	5 (3.3%)	16 (2.6%)	5 (6.3%)	16 (4.0%)	0.106
MACCE at 1 year (%)	24 (15.7%)	83 (13.5%)	16 (20.0%)	63 (15.6%)	0.147
Mortality (%)	2 (1.3%)	9 (1.5%)	4 (5.0%)	6 (1.5%)	0.266
Hard clinical endpoint (mortality/AMI/stroke) (%)	3 (2%)	19 (3.1%)	7 (8.8%)	16 (4.0%)	0.058
Target vessel revascularization (%)	21 (13.7%)	66 (10.7%)	11 (13.8%)	54 (13.3%)	0.159

*AMI, acute myocardial infarction; MACCE, major adverse cardiac and cerebrovascular events*.

Table 4 provides the age-adjusted HR for women and men in the treatment group compared to control. Overall, there was no significant interaction of group allocation and sex.

**Table 4:** Clinical safety outcome results in cell therapy groups stratified by sex.

**Clinical endpoints**		***P* for interaction**^#^
In-hospital complications, adjOR		
Men in treatment group	0.94 (−0.76–3.86)	0.64
Women in treatment group	−0.94 (−3.86–0.76)	
MACCE at 1 year, adjHR		
Men in treatment group	0.88 (0.56–1.4)	0.96
Women in treatment group	1.13 (0.72–1.79)	
Mortality, adjHR		
Men in treatment group	1.47 (0.31–6.89)	0.21
Women in treatment group	0.68 (0.15–3.2)	
Hard clinical endpoint (mortality/AMI/stroke), adjHR		
Men in treatment group	1.9 (0.56–6.48)	0.13
Women in treatment group	0.53 (0.15–1.79)	
Target vessel revascularization, adjHR		
Men in treatment group	0.78 (0.79–2.11)	0.43
Women in treatment group	1.29 (0.47–1.27)	

*Primary safety endpoint;

# *p for interaction of gender with group allocation; adjHR, hazard ratio adjusted for age; adjOR, odds ratio adjusted for age; AMI, acute myocardial infarction; MACCE, major adverse cardiac and cerebrovascular events*.

### Efficacy Endpoints

Women had a significantly smaller EDV and ESV and a higher EF than men (50.1 ± 14.2% vs. 47.2 ± 13.6%, respectively; *p* = 0.010) at follow-up. Additionally, the post-infarction increase in EDV was significantly lower in women than in men (9.4 ± 30.5 vs. 15.6 ± 38.7 mL; *p* = 0.0482) (Table 5). Moreover, women had a numerically higher but not statistically significant increase in delta-EF.

**Table 5:** Follow-up left ventricular functional data efficacy endpoints stratified by sex.

	**Women**	**Men**	***P*-value**
	**(*n* = 233)**	**(*n* = 1,019)**	
Follow-up EDV (mL)	125.9 ± 43.1	165.1 ± 55.9	<0.001
Follow-up ESV (mL)	66.4 ± 36.9	90.2 ± 47.3	<0.001
Follow-up EF (%)	50.1 ± 14.2	47.2 ± 13.6	0.010
Delta EDV (mL)	9.4 ± 30.5	15.6 ± 38.7	0.048
Delta ESV (mL)	2.8 ± 27.2	5.2 ± 31.1	0.348
Delta EF (%)^*^	4.1 ± 10.6	3.0 ± 9.0	0.154
Indexed EDV^#^	−0.029 (−0.196, 0.084)	−0.078 (−0.257, 0.057)	0.059
Indexed ESV^#^	0.021 (−0.282, 0.212)	−0.031 (−0.263, 0.171)	0.136
Indexed EF^#^	0.111 ± 0.279	0.086 ± 0.251	0.220

* *Primary efficacy endpoint*.

# *indexing was performed by dividing the delta values by their baseline values. EDV, end-diastolic volume; EF, ejection fraction; ESV, end-systolic volume*.

In the subgroup analysis, women in both groups had a significantly lower follow-up EDV and ESV and a numerically higher (non-significant) follow-up EF (Supplementary Table 3). Cell-treated women had a numerically higher increase in EF as compared to women in the control group and men in both groups (Supplementary Table 3).

**Figure 2:**
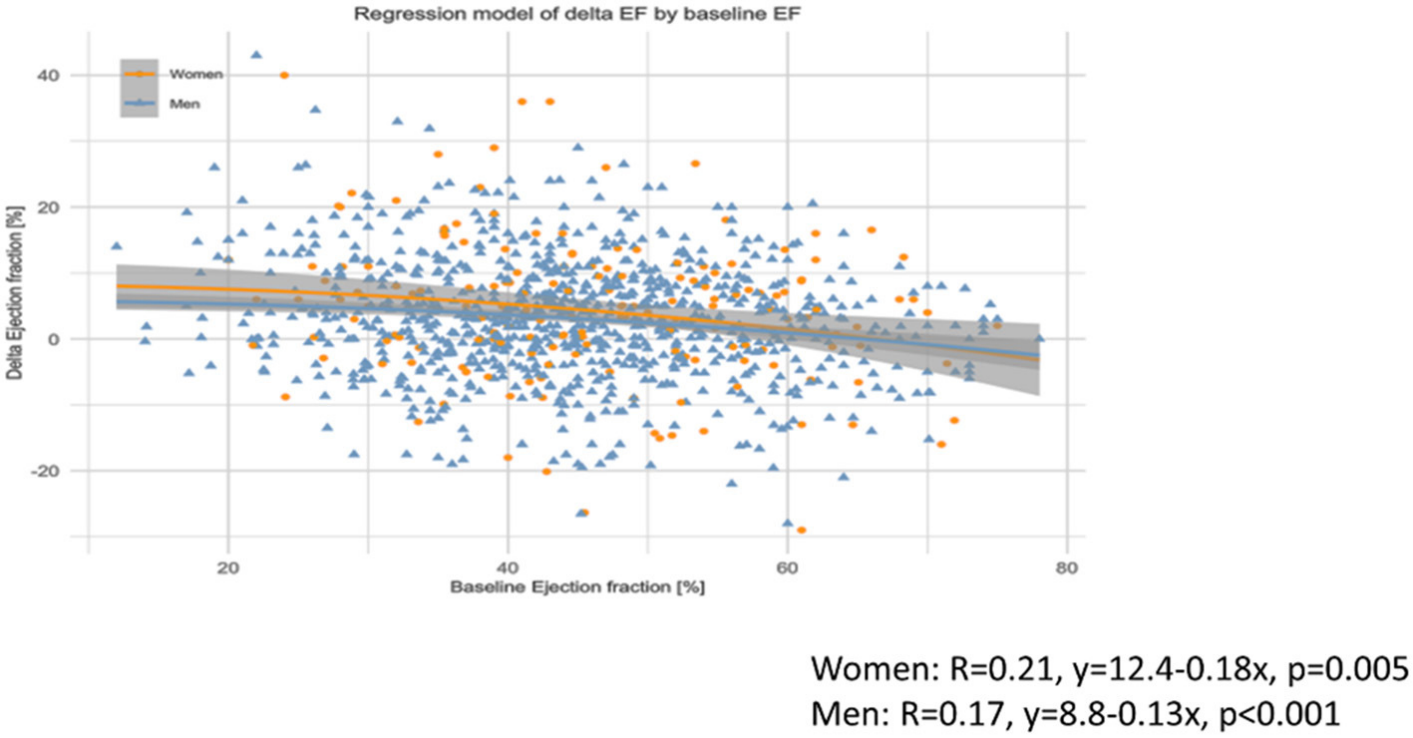
Association between baseline ejection fraction (EF) and changes in EF in women and men. Significant but very weak negative correlation between baseline EF and changes in EF both in women and men, with no difference between the sexes.

Regression analysis revealed a significant negative association between baseline EF and changes in EF in both men and women, but with a very low regression coefficient (*R* ≤ 0.21) (Figure 2); similar results were seen if the correlation between pre-EF and changes in EF was separately plotted in women or men of the cell therapy or controls groups, respectively (*R* ≤ 0.17, data not shown).

### Sub-analysis of Bone-Marrow-Derived Cells and Baseline Characteristics and Outcomes

**Figure 3:**
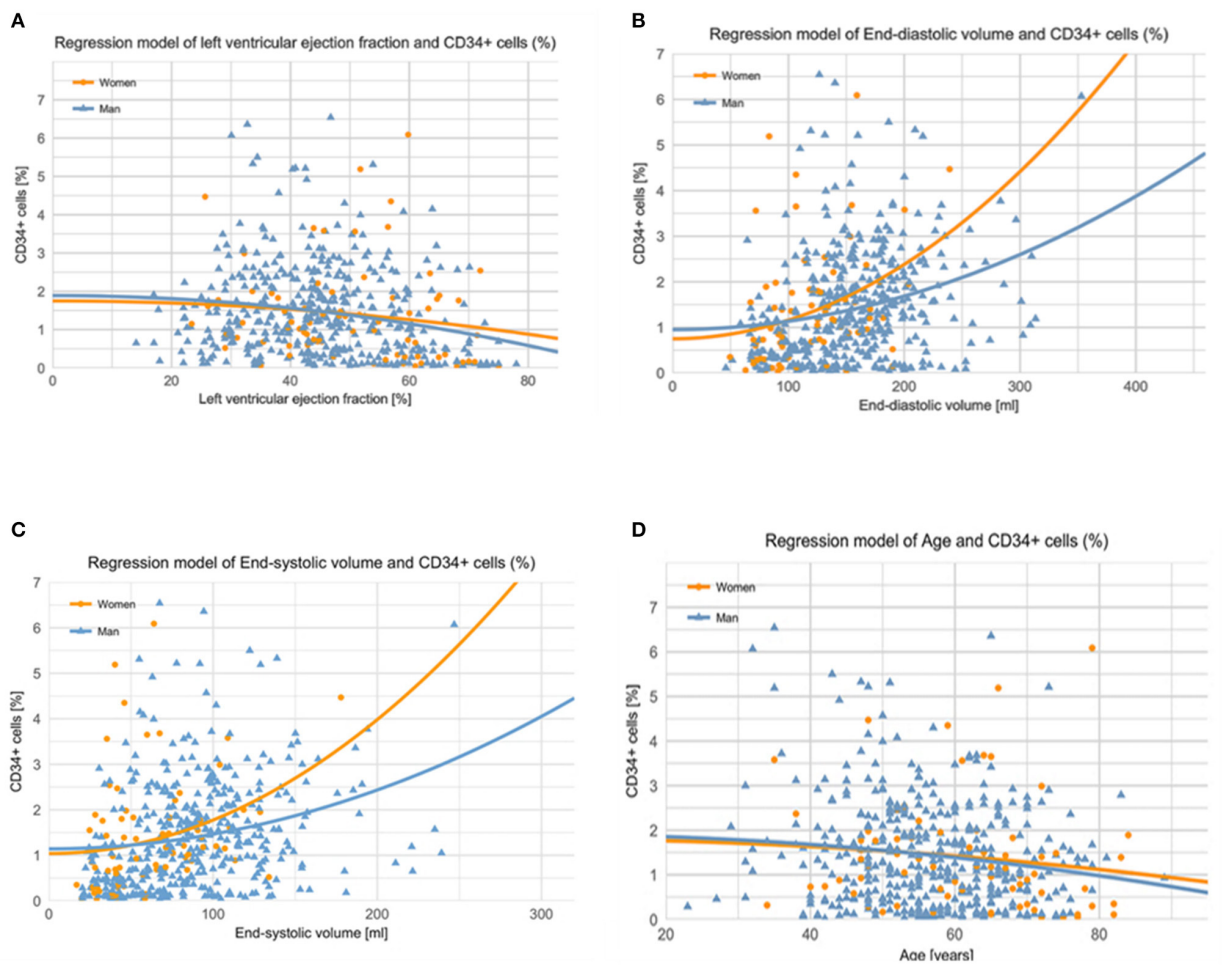
**(A)** Correlation of baseline left ventricular ejection fraction with CD34+ cells. Left ventricular ejection fraction (EF) values correlated negatively with the percentage of CD34+ cells. The higher the EF is, the lower the number of BM-origin CD34+ cells (in percentage) in both sexes. The results were similar in men and women. Men: *r* = 0.282; y (lnCD34posPercent) = 2.81–0.025xEF (men); *p* < 0.001. Women: *r* = 0.32; y (lnCD34posPercent) = 3.547–0.029xEF (women); *p* = 0.001. **(B)** End-diastolic volume (EDV) values showed a significant exponential correlation with the percentage of CD34+ cells, suggesting an intrinsic repair mechanism in both sexes. The left shift of the curve in women indicates that a smaller EDV value was associated with a higher percentage of CD34+ cells in women. Men: *r* = 0.26; y (lnCD34posPercent) = 0.351+0.006xEDV (men); *p* < 0.001. Women: *r* = 0.362; y (lnCD34posPercent) = 0.243+0.011xEDV (women); *p* = 0.002. **(C)** Correlation of baseline end-systolic volume with CD34+ cells. End-systolic volume (ESV) showed an exponential correlation with the number of BM-origin CD34+ cells (in percentage). The left shift of the curve in women indicates that a smaller ESV results in a higher percentage of CD34+ cells in women as compared to men. Men: *r* = 0.316; y (lnCD34posercent) = 0.406+0.009xESV (men); *p* < 0.001. Women: *r* = 0.407; y (lnCD34posPercent) = 0.334+0.015xESV (women); *p* < 0.001. **(D)** Correlation of patient age with CD34+ cells. Age was negatively, weakly correlated with CD34+ cells in women, with a non-significant correlation in men. The lower the age of women, the higher the number of BM-origin CD34+ cells [in percentage]. Men: *r* = 0.094; y (lnCD34posPercent) = 1.60–0.01xAge (men); *p* = 0.054. Women: *r* = 0.285; y (lnCD34posPercent) = 4.52–0.027xAge (women); *p* = 0.013.

Table 2 shows the percentage of BM-CD34+ hematopoietic progenitor cells and BM-CD34+CD133+ endothelial progenitor cells at baseline as stratified by sex. In the multivariable covariance analysis, the frequency of CD34+ hematopoietic progenitor cells in the BM in both sexes was positively associated with baseline EDV and ESV values and negatively associated with EF (Table 6 and Figures 3A–C).

**Table 6:** Percentage of CD34+ cells in women and men with different comorbidities.

**Comorbidity**	**Sex**	**Percentage of CD34 + cells(Mean ± SD)**	***P*-value**
Diabetes mellitus			
Yes (*n* = 69)	Men (*n* = 62)	1.67 ± 1.3	0.600
	Women (*n* = 7)	2.01 ± 1.56	
No (*n* = 403)	Men (*n* = 335)	1.39 ± 1.39	0.681
	Women (*n* = 68)	1.33 ± 1.33	
Hypertension			
Yes (*n* = 227)	Men (*n* = 189)	1.54 ± 1.14	0.343
	Women (*n* = 38)	1.34 ± 1.3	
No (*n* = 266)	Men (*n* = 229)	1.35 ± 1.22	0.672
	Women (*n* = 37)	1.44 ± 1.23	
Smoking			
Yes (*n* = 251)	Men (*n* = 222)	1.63 ± 1.63	0.785
	Women (*n* = 29)	1.56 ± 1.33	
No (*n* = 197)	Men (*n* = 162)	1.33 ± 1.12	0.334
	Women (*n* = 35)	1.53 ± 1.29	
EDV in quartiles (mL)			<0.001
1. quartile (*n* = 107)	Men (*n* = 71)	0.75 ± 0.68	
(46.1–114.8 mL)	Women (*n* = 36)	1.07 ± 1.26	
2. quartile (*n* = 101)	Men (*n* = 82)	1.32 ± 1.36	
(114.9–148.3 mL)	Women (*n* = 18)	1.42 ± 0.64	
3. quartile (*n* = 128)	Men (*n* = 115)	1.55 ± 1.14	
(148.4–181.0 mL)	Women (*n* = 13)	1.90 ± 1.9	
4. quartile (*n* = 147)	Men (*n* = 140)	1.78 ± 1.2	
(181.1–352.9 mL)	Women (*n* = 7)	1.98 ± 1.57	
ESV in quartiles (mL)			<0.001
1. quartile (*n* = 121)	Men (*n* = 83)	0.78 ± 0.84	
(16.9–53.0 mL)	Women (*n* = 37)	1.12 ± 1.23	
2. quartile (*n* = 109)	Men (*n* = 102)	1.44 ± 1.3	
(53.1–80.0 mL)	Women (*n* = 18)	1.68 ± 1.5	
3. quartile (*n* = 115)	Men (*n* = 108)	1.65 ± 1.15	
(80.1–106.4 mL)	Women (*n* = 11)	1.35 ± 0.63	
4. quartile (*n* = 138)	Men (*n* = 115)	2.02 ± 1.36	
(106.5–246.8 mL)	Women (*n* = 8)	1.73 ±1.19	
Ejection fraction (in quartiles) (%)			<0.001
1. quartile (*n* = 124)	Men (*n* = 107)	1.51 ± 1.21	
(14.04–35.78%)	Women (*n* = 12)	1.53 ± 1.26	
2. quartile (*n* = 96)	Men (*n* = 103)	1.65 ± 1.17	
(35.79–44.98%)	Women (*n* = 17)	1.29 ± 0.8	
3. quartile (*n* = 116)	Men (*n* = 103)	1.60 ± 1.22	
(44.99–56.0%)	Women (*n* = 17)	1.68 ± 1.32	
4. quartile (*n* = 147)	Men (*n* = 95)	0.95 ± 1.0	
(56.01–78.9 %)	Women (*n* = 28)	1.22 ± 1.49	
Age (in quartiles) (years)			0.826
quartile (*n* = 124) (23–50)	Men (*n* = 107)	1.43 ± 1.44	
	Women (*n* = 16)	1.53 ± 1.15	
2. quartile (*n* = 96) (51–57)	Men (*n* = 112)	1.41 ± 1.12	
	Women (*n* = 12)	1.49 ± 0.76	
3. quartile (*n* = 116) (58–65)	Men (*n* = 110)	1.29 ± 1.07	
	Women (*n* = 18)	1.63 ± 1.32	
4. quartile (*n* = 147) (66–89)	Men (*n* = 76)	1.23 ± 0.96	
	Women (*n* = 28)	1.16 ± 1.45	

*EDV, end-diastolic volume; ESV, end-systolic volume; SD, standard deviation*.

Regression analysis showed a left shift of the curve in women indicating a higher percentage of BM-CD34+ hematopoietic progenitor cells in women than in men with similar-sized left ventricles (Figures 3A–C). In men, we found no association between age and frequency of CD34+ hematopoietic progenitor cells in the BM; however, in women, younger age was associated with a higher percentage of BM-CD34+ hematopoietic progenitor cells (Figure 3D).

**Figure 4:**
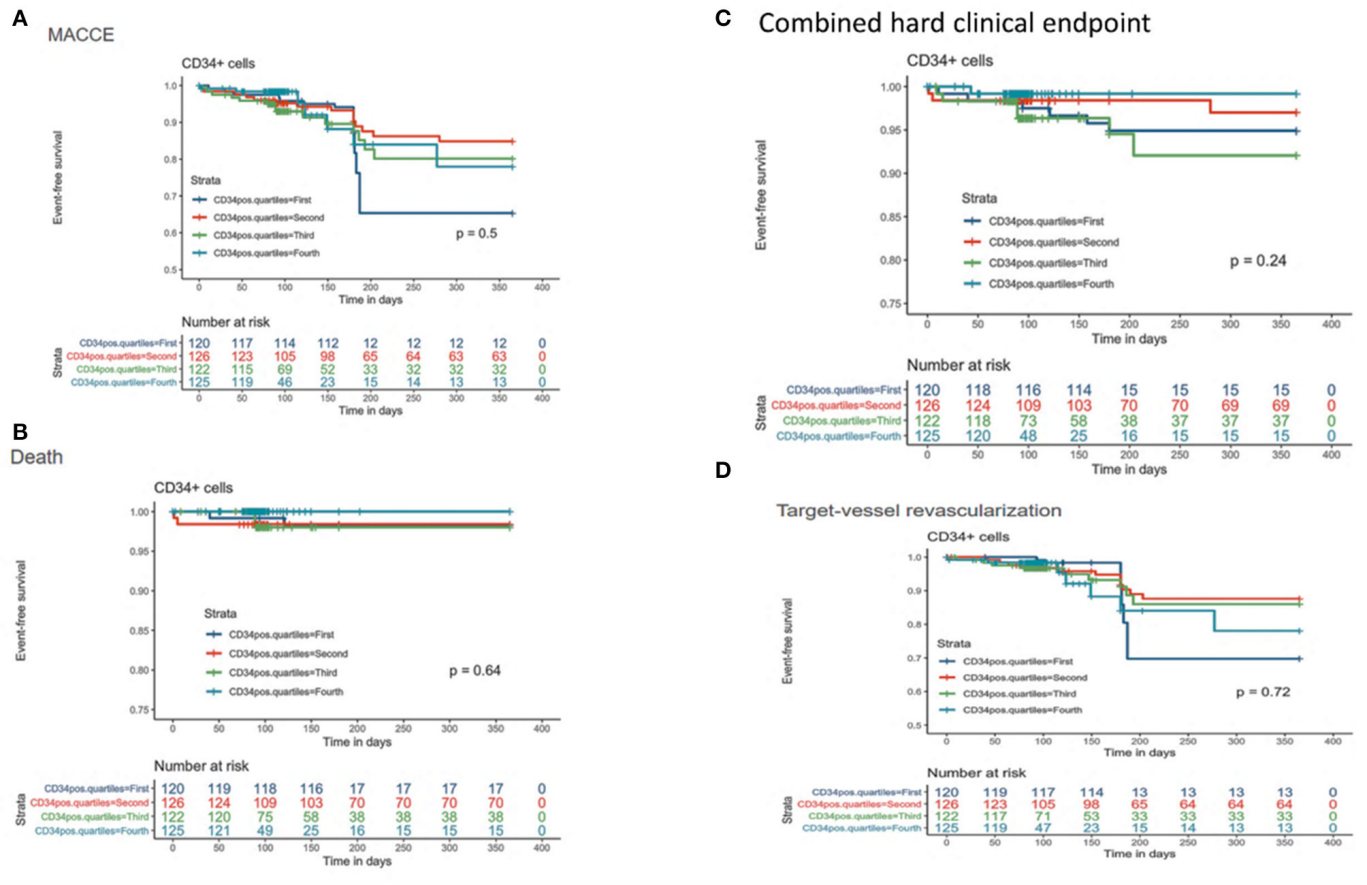
Kaplan-Meier curves for CD34+ cells stratified by quartiles and clinical outcomes. **(A)** Major adverse cardiac and cerebrovascular event(s) (MACCE). **(B)** Death. **(C)** Composite endpoint (mortality, stroke, acute myocardial infarction). **(D)** Target-vessel revascularization.

**Figure 5:**
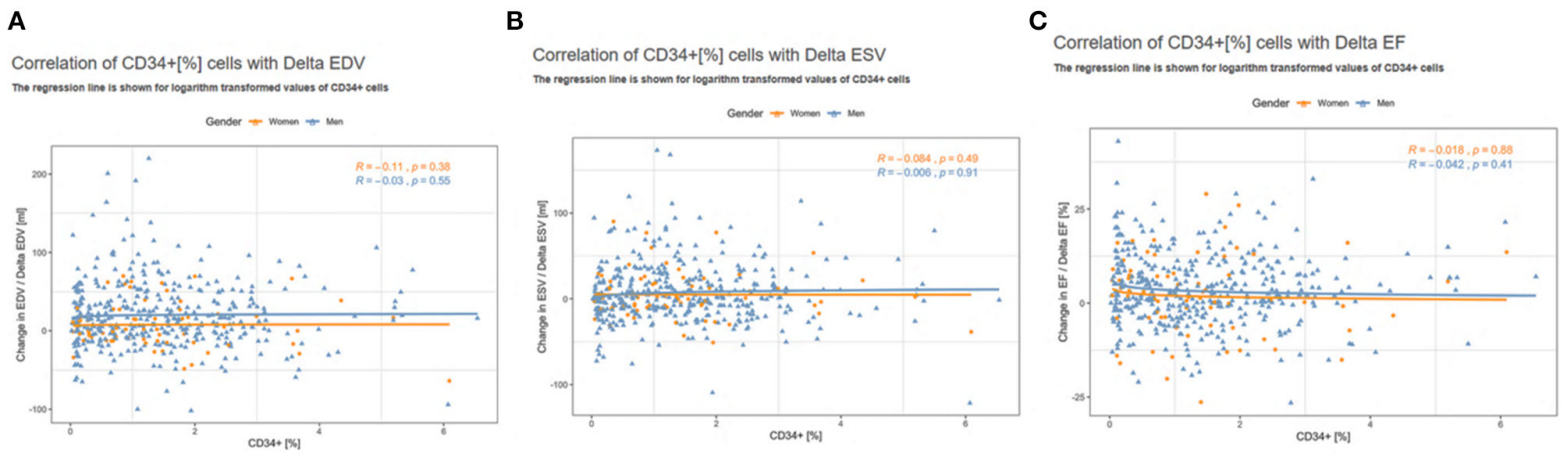
Correlation of CD34+ cells left ventricular functional data. **(A)** Delta EDV. **(B)** Delta ESV. **(C)** Delta EF. The regression line is shown for logarithmic transformed values of bone marrow CD34+ cells.

No association was found between BM-CD34+ cells and any clinical endpoint (Figure 4 and Supplementary Table 1); no sex-related sub-analysis was performed because of the small number of women in subgroups and the lack of a significant effect of the number of BM-CD34+ cells on outcomes. In addition, we found no association between the number of BM-CD34+ cells and any of the following cardiac variables: delta-ESV, EDV, and EF (Figure 5). Similarly, the number of BM-CD34+133+ cells did not correlate with any of the endpoints (data not shown).

## Discussion

Women with cardiovascular disease are an underdiagnosed, undertreated, and under-investigated population despite increasing public awareness (19, 20) and initiatives designed to reduce sex disparities in clinical trials of cardiovascular therapies (21). In this analysis of a collaborative, multinational database of patients with ischemic heart disease enrolled in 12 cell-therapy trials, sex-based differences in baseline characteristics and outcomes were identified, despite the limited number of women enrolled in the trials. To our knowledge, this is the first report of differences in baseline characteristics and outcomes by sex in cell-based therapy in an AMI cohort. In this *post-hoc* analysis of the ACCRUE dataset, we found that women represented <20% of all enrolled patients in cardiovascular cell therapy trials for AMI. Our findings are consistent with previous reports showing a low number of women enrolled in cardiac studies (21, 22). The underrepresentation of women decreases the power of clinical trials to yield data on sex-specific conclusions and limits the recognition of sex-based differences, thus preventing the optimization of therapy for women (7). The reason for the lower number of women enrolled in these 12 cell-based cardiac studies is unknown but may relate to the findings that women are less likely than men to seek medical assistance for cardiovascular diseases and are older when AMI or heart failure first manifests (4, 23). The Cardiovascular Cell Therapy Research Network reported that the influence of a family member and an unwillingness to receive placebo were the reasons most often cited by women for not enrolling in or for withdrawing from cell therapy studies (24).

### Sex-Based Differences in Baseline Characteristics

Our analysis showed that lifestyle risk factors and comorbidities varied by sex. Cigarette smoking has decreased overall in the United States, but we found that smoking was more common in men than in women (Table 1). In our study, women were older and had a greater prevalence of hypertension. In previous reports, the prevalence of hypertension has been lower in young women than in men, but that trend reverses in older women (>60 years) (4, 25). Hypertension has been associated with endothelial dysfunction and atherosclerosis (26, 27). In the Framingham study, 75% of new heart failure patients had hypertension. In individuals with hypertension, LV afterload is chronically increased, and EF is decreased (28). Over the long term, the chronic increase in workload triggers adverse myocardial remodeling and dysfunction (29). Dash et al. showed that male mice exhibited increased activation of P38 MAPK, which plays a role in cardiac remodeling/hypertrophy. Moreover, this P38 MAPK activation occurred in acyclic female mice (30). In our study, EF was preserved from baseline to follow-up in women compared with men, despite the higher rates of hypertension in women. This finding may suggest an increased tolerance to a high cardiac workload in women or may simply indicate a higher incidence of preserved ejection fraction despite cardiac injury (15).

Additionally, we found marked differences in cardiac function between the sexes. Women had a lower EDV and ESV and a numerically higher EF at baseline than did men. This finding supports previous data that show EF is typically higher in women, even after AMI (7, 11). Several groups have reported that the adaptation to pressure overload and stress differs between men's and women's hearts ADDIN EN.CITE (6, 31, 32). Concentric myocardial hypertrophy induced by pressure overload in women is associated with a smaller internal chamber and a relatively thicker wall in postmenopausal women, whereas eccentric hypertrophy is chiefly seen in men (33).

Despite randomization in these clinical trials, our analysis showed that women in the control group differed from those in the cell therapy cohort: The former had a higher EF, lower peak CK values, and a smaller ESV. In contrast, men in the control group had a smaller EDV and ESV but similar CK values when compared with cell-treated men, supporting the published ACCRUE results (16).

### Sex-Based Differences in Outcomes

Because of improvements in the treatment and prevention of AMI, mortality from coronary artery disease has significantly decreased over the past decades (34). However, this reduction in mortality has been less pronounced in women than in men (17). In a multicenter study, being female was an independent predictor for re-hospitalization in patients with acute coronary syndrome (35). Interestingly, in our study, we demonstrated that women in the control group had a higher composite hard clinical endpoint (stroke, AMI, and mortality) than cell-treated women. Several groups have shown that the cardiovascular mortality rate is substantially higher in women than in men (12). We found this was true only in women in the control group, despite the fact that women overall had a higher prevalence of hypertension and were older.

### Correlation of BM-CD34+ Cells With Patient Sex

Sex-related differences have been found in the potency of stem cells. Payne et al. reported that the chondrogenic differentiation of human BM-derived stem cells declined with age in men but not in women (36). In preclinical studies, BM-derived mesenchymal stem cells (MSCs) from newborn female Sprague-Dawley rats had a greater therapeutic efficacy than MSCs from newborn male rats in reducing neonatal hyperoxia-induced inflammation and vascular remodeling, suggesting that MSCs from females may be more potent at repair than MSCs from males (37). Stem cells are sensitive to hormones in the local environment including sex hormones (38). Increasing evidence indicates that hormonal signals can critically influence stem cell function (39). In a study of women who were twins, long-term use of hormone replacement therapy was associated with better muscle composition and performance orchestrated by improved regulatory actions on the cytoskeleton and the intramuscular extracellular matrix and with a switch from glucose-oriented metabolism to fatty acids (40). Thus, female-specific biologic factors may be at play. However, in contrast with the promising pre-clinical small animal data (41–43) our study did not show a robust effect of cell therapy in women, most probably because the vast majority of the women were in postmenopausal age with supposed decrease in circulating estradiol level (44–47).

CD34+ cells are also found in the circulation and can be differentiated into hematopoietic, endothelial, and non-hematopoietic lineages and can promote angiogenesis (48) and cardiac repair after injury (49, 50). The frequency of CD34+ cells in the BM is used as an indicator of BM potency in the transplant field, and these cells (51–53) have been shown to repair the damage associated with CVD. Furthermore, CD34+ cell therapy has been associated with positive outcomes in patients with refractory angina, and the frequency of circulating CD34+ cells has been associated with a decreased risk of future cardiovascular events (54).

Recently, the frequency of circulating CD34+ cells was shown to differ by race (48). In addition, CD34+ hematopoietic stem cells have been found to express sex-related hormone receptors that changed functions in *in-vitro* studies (55, 56). Here, we found no differences in the percentage of BM-CD34+ cells between men and women, but we did show that the frequency of CD34+ cells was negatively associated with age in women but not in men (Figure 3D). Moreover, CD34+ cell frequency was positively associated with ESV and EDV and negatively associated with EF in both men and women. Increased age is a major contributor to endothelial dysfunction (57) and cardiovascular risk. Perin et al. (58) reported that in studies of BM cell therapy for heart failure, LVEF improvement was positively correlated with the percentage of BM-CD34+ cells, in contrast with our study of patients with AMI.

### Study Limitations

Concomitant valvular diseases, e.g., severe mitral insufficiency of ischemic origin could considerably affect the results. However, severe valve diseases were exclusion criteria in all trials, and no necessary heart valve surgery was reported in the included studies. LV wall thickness and its changes during the follow-up measured by echocardiography would be a good additional parameter to evaluate the LV functional parameter. However, measurements of this parameter can be misleading in different infarct wall locations and was not included into the harmonized ACCRUE database.

Since women in our study have smaller hearts, adjusting of the LV parameter with BMI would be reasonable, even if no sex difference according to BMI was found in a meta-analysis including 12 million people with coronary heart disease (59). Unfortunately, the ACCRUE database does not contain BMI data. However, EDV, ESV and EF indexes were not different between man and women (Table 5).

This is a *post-hoc* secondary analysis from prospective, randomized, controlled clinical trials, included in the ACCRUE database; therefore, the findings should be considered hypothesis-generating.

## Conclusions

Our results provide further evidence that women are underrepresented in studies of cell therapy after AMI. Moreover, we show that patient sex may affect cardiac function and the clinical response to autologous cell therapy. These differences should be considered in cardiac reparative studies where the composition of the product is patient dependent and where findings may be confounded by mixing data by sex. Finally, these sex-specific findings, although exploratory, reflect the best data available concerning autologous cell therapy in patients after AMI. Reporting sex differences is critical to allow more in-depth analysis by multiple groups across the regenerative medicine spectrum. Further analyses are warranted to better understand the biological processes of sex differences in cardiovascular diseases and in sex-based responses to regenerative medicine therapies.

## Data Availability Statement

The data analyzed in this study is subject to the following licenses/restrictions: Requests to access these datasets should be directed to Mariann Gyöngyösi, mariann.gyongyosi@meduniwien.ac.at.

## Author Contributions

PH performed analysis and interpretation of data, drafting and revising the manuscript critically for important intellectual content, statistical analysis, tables, figures, and contributed to analysis plan and manuscript revision. MG, LC-A, CH-M, LS, and DT performed analysis and interpretation of data, drafting and revising the manuscript critically for important intellectual content, and manuscript revision. DT performed idea conceptualization and convening the study group. All authors contributed to the article and approved the submitted version.

## Conflict of Interest

DT is self-employed as a business consultant with RegenMedix Consulting LLC. At the time the work was performed DT was employed by Texas Heart Institute. The remaining authors declare that the research was conducted in the absence of any commercial or financial relationships that could be construed as a potential conflict of interest.
