# Supplementary material for: Sex-Based Differences in Autologous Cell Therapy Trials in Patients With Acute Myocardial Infarction: Subanalysis of the ACCRUE Database
Source: Front Cardiovasc Med. 2021 May 26;8:664277. doi: 10.3389/fcvm.2021.664277 (PMC8187782; doi:10.3389/fcvm.2021.664277)
Supplement: Supplementary file 1 [file Data_Sheet_1.PDF]

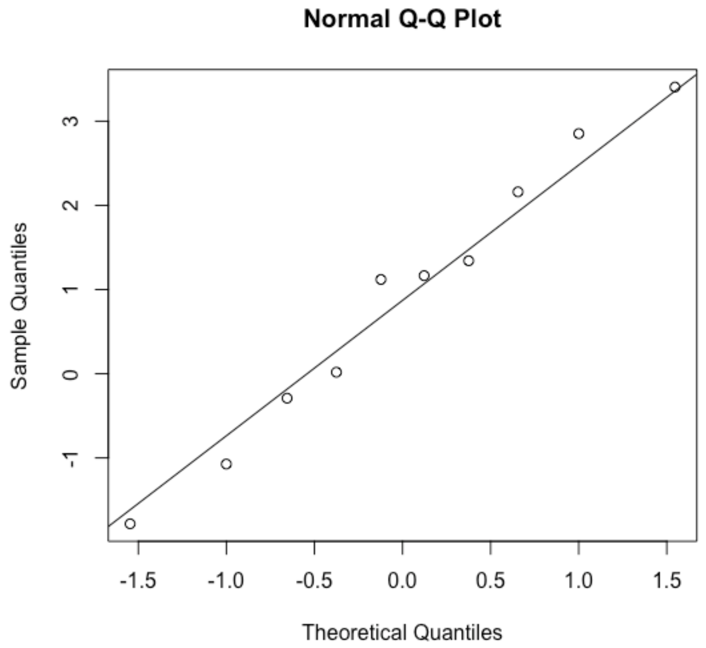

Q-Q plot with n= 10, Shapiro Wilk p-value = 0.9119

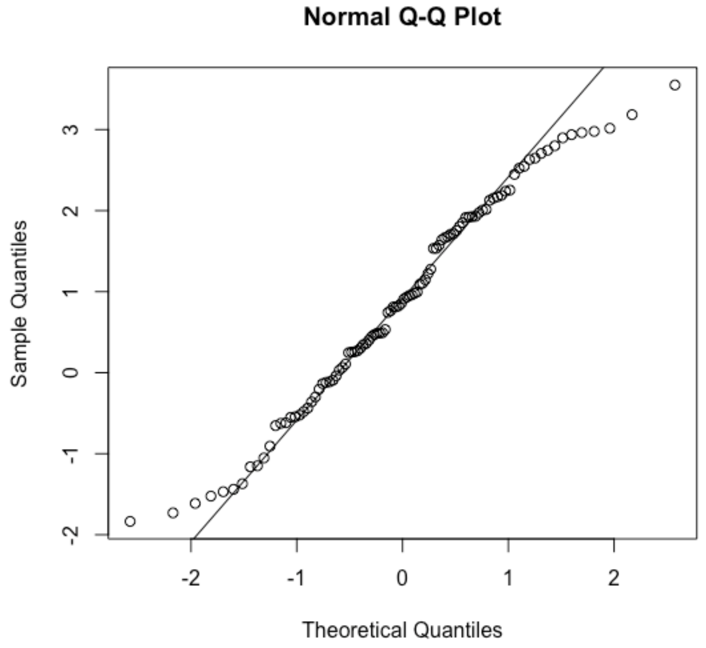

Q-Q plot with n= 100, Shapiro Wilk p-value = 0.09479

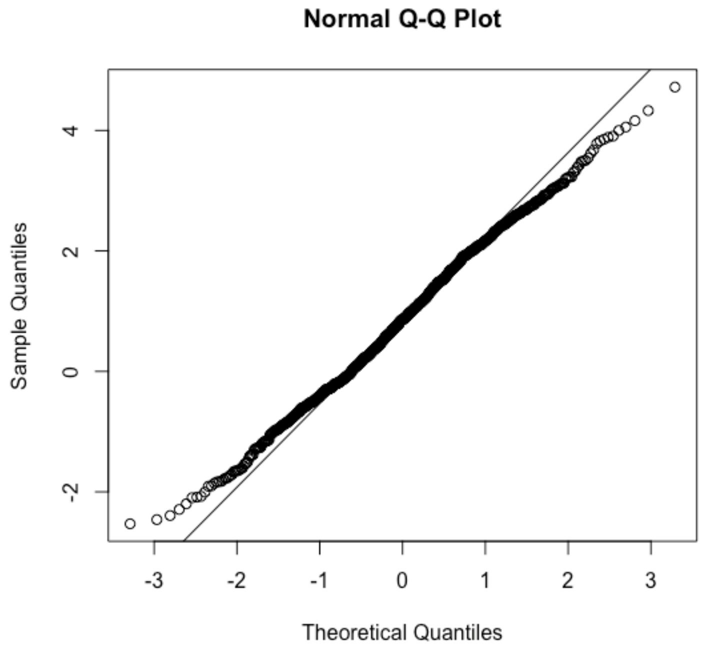

Q-Q plot with n= 1000, Shapiro Wilk p-value = 0.02461 => significant !

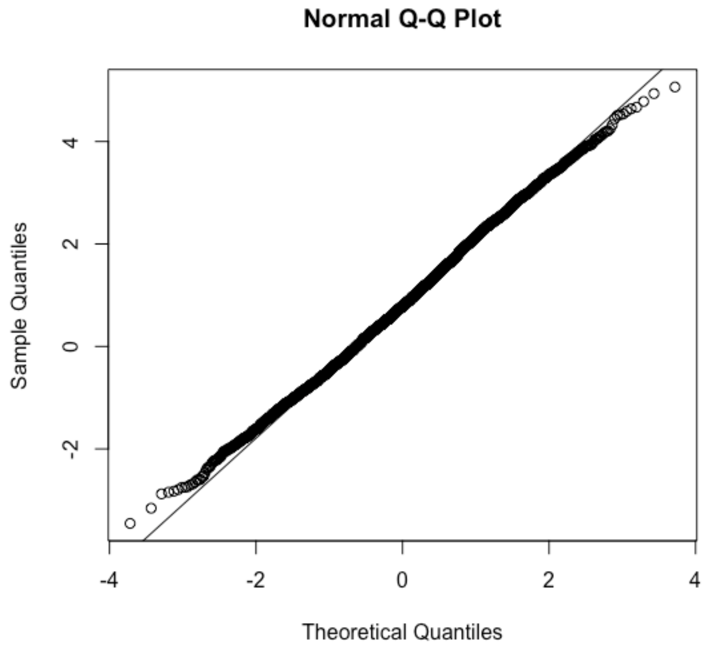

Q-Q plot with n= 5000, Shapiro Wilk p-value = 0.0007863 => significant !

In this context and with respect to our data we still think that the assumption of normality holds for the analyzed data in the present manuscript.

R code:

```
### Generate 100 replicates of random data with almost normal distribution for artificial data sets
of varying size (n=10, 100, 1000, 5000)

x <- replicate(100, { # generates 100 different tests on each distribution
  c(shapiro.test(rnorm(10)+c(1,0,2,0,1))$p.value,   #$
    shapiro.test(rnorm(100)+c(1,0,2,0,1))$p.value,  #$
    shapiro.test(rnorm(1000)+c(1,0,2,0,1))$p.value, #$
    shapiro.test(rnorm(5000)+c(1,0,2,0,1))$p.value) #$
} # rnorm gives a random draw from the normal distribution
)

### provide rownames for the results of Shapiro Wilk replications
rownames(x) <- c("n10","n100","n1000","n5000")

### provide the proportion of significant deviations according to Shapiro Wilk
rowMeans(x<0.05)
x

### generate 4 examples for each size of dataset
x1 <- rnorm(10)+c(1,0,2,0,1)
x2 <- rnorm(100)+c(1,0,2,0,1)
x3 <- rnorm(1000)+c(1,0,2,0,1)
x4 <- rnorm(5000)+c(1,0,2,0,1)

### generate four qqplots for the four different artificial datasets
qqx1 <- qqnorm(x1); qqline(x1)
qqx2 <- qqnorm(x2); qqline(x2)
qqx3 <- qqnorm(x3); qqline(x3)
qqx4 <- qqnorm(x4); qqline(x4)

### provide the individual Shapiro Wilk results for the specific four datasets
shapiro.test(x1)
shapiro.test(x2)
shapiro.test(x3)
shapiro.test(x4)
```
