# Supplementary material for: Sex-Based Differences in Autologous Cell Therapy Trials in Patients With Acute Myocardial Infarction: Subanalysis of the ACCRUE Database
Source: Front Cardiovasc Med. 2021 May 26;8:664277. doi: 10.3389/fcvm.2021.664277 (PMC8187782; doi:10.3389/fcvm.2021.664277)
Supplement: Supplementary file 2 [file Data_Sheet_2.docx]

Supplementary Material

**Supplementary Table 1.** Baseline characteristics in the cell-treated group stratified by sex.

| **Cell-therapy group** | **Women** | **Men** | **P value** |
| --- | --- | --- | --- |
|  | (n=153) | (n=614) |  |
| Age (years)* | 60.0 ± 11.2 | 56.7 ± 10.1 | < 0.001 |
| Hypertension (%) | 86 (57.7) | 298 (48.6) | 0.046 |
| Hyperlipidemia (%) | 79 (56.0) | 308 (54.0) | 0.670 |
| Smoking (%) | 64 (47.4) | 332 (58.3) | 0.021 |
| Diabetes (%) | 23 (15.4) | 88 (14.4) | 0.737 |
| Number of diseased vessels (%) | |  | 0.016 |
| 1, n (%) | 129 (86.6%) | 468 (76.3%) |  |
| 2, n (%) | 11 (7.4%) | 98 (16.0%) |  |
| 3, n (%) | 9 (6.0%) | 47 (7.7%) |  |
| Maximum CK (U/L)* | 2720.0 ±2351.7 | 3281.3 ± 2445.8 | 0.034 |
| Vessel target LAD (%) | 136 (89.5) | 527 (85.8) | 0.239 |
| Pre-EDV (mL)* | 121.1 ± 40.2 | 152.1 ± 51.3 | < 0.001 |
| Pre-ESV (mL)* | 68.5 ± 32.1 | 87.4 ± 40.3 | < 0.001 |
| Pre-EF (%)* | 44.2 ± 12.4 | 43.5 ± 11.8 | 0.567 |
| % CD34+ (n=493) (%) | 1.3 ± 1.2 | 1.3 ± 1.1 | 0.853 |
| % CD34+CD133+ (n=206) (%) | 1.1 ± 0.8 | 1.1 ± 0.6 | 0.897 |

*Mean ± SD. CK, creatine kinase; EDV, end-diastolic volume; EF, ejection fraction; ESV, end-systolic volume; LAD, left anterior descending artery.

**Supplementary Table 2.** Baseline characteristics in the control group stratified by sex.

| **Control group** | **Women** | **Men** | **P value** |
| --- | --- | --- | --- |
|  | **(n=80)** | **(n=405)** |  |
| Age (years)* | 61.1 ± 9.7 | 56.2 ± 10.7 | < 0.001 |
| Hypertension (n) | 45 (56.2) | 199 (49.1) | 0.245 |
| Hyperlipidemia (n) | 37 (51.4) | 191 (52.6) | 0.849 |
| Smoking (n) | 36 (51.4) | 207 (58.8) | 0.254 |
| Diabetes (n) | 11 (13.8) | 68 (16.8) | 0.501 |
| Number of diseased vessels (n) |  |  | 0.177 |
| 1, (%) | 65 (82.3) | 295 (75.8) |  |
| 2, (%) | 8 (10.1) | 72 (18.5) |  |
| 3, (%) | 6 (7.6) | 22 (5.7) |  |
| Maximum CK (n)* | 2184 ± 1464 | 3154 ± 2356 | 0.002 |
| Vessel target LAD (n) | 1 (100.0) | 22 (95.7) | 0.831 |
| Pre-EDV (mL)* | 112.8 ± 36.2 | 144.6 ± 46.6 | < 0.001 |
| Pre-ESV (mL)* | 59.9 ± 25.2 | 81.1 ± 36.4 | < 0.001 |
| Pre-EF (%)* | 47.9 ± 11.2 | 45.0 ± 11.9 | 0.055 |

*Mean ± SD. CK, creatine kinase; EDV, end-diastolic volume; EF, ejection fraction; ESV, end-systolic volume; LAD, left anterior descending artery.

**Supplementary Table 3** Efficacy endpoints in cell-treated and controls stratified by sex.

| **Groups** | **Women** | **Men** | **P value** |
| --- | --- | --- | --- |
|  |  |  |  |
|  | **(n=80)** | **(n=504)** |  |
| **Control** |  |  |  |
| Follow-up EDV (n) | 121.0 ± 39.9 | 158.9 ± 53.3 | < 0.001 |
| Follow-up ESV (n) | 61.1 ± 31.1 | 85.7 ± 44.7 | < 0.001 |
| Follow-up EF (%) | 51.0 ± 12.8 | 47.8 ± 13.5 | 0.0752 |
| Delta EDV (n) | 11.4 ± 30.4 | 14.4 ± 34.0 | 0.5052 |
| Delta ESV (n) | 3.4 ± 23.0 | 4.8 ± 28.1 | 0.6932 |
| Delta ejection fraction (n) | 2.8 ± 11.1 | 2.6± 8.5 | 0.8122 |
| **Cell therapy** | (n=153) | (n=614) |  |
| Follow-up EDV (n) | 128.8 ± 44.8 | 169.7 ± 57.4 | < 0.005 |
| Follow-up ESV (n) | 69.4 ± 39.7 | 93.5 ± 48.9 | < 0.005 |
| Follow-up EF (n) | 49.5 ± 15.0 | 46.8 ± 13.6 | 0.0542 |
| Delta EDV (n) | 8.3 ± 30.6 | 16.4 ± 41.7 | 0.0542 |
| Delta ESV (n) | 2.5 ± 29.4 | 5.5 ± 33.1 | 0.3872 |
| Delta EF (n) | 4.7 ± 10.3 | 3.3 ± 9.3 | 0.1352 |

EDV, end-diastolic volume; EF, ejection fraction; ESV, end-systolic volume.


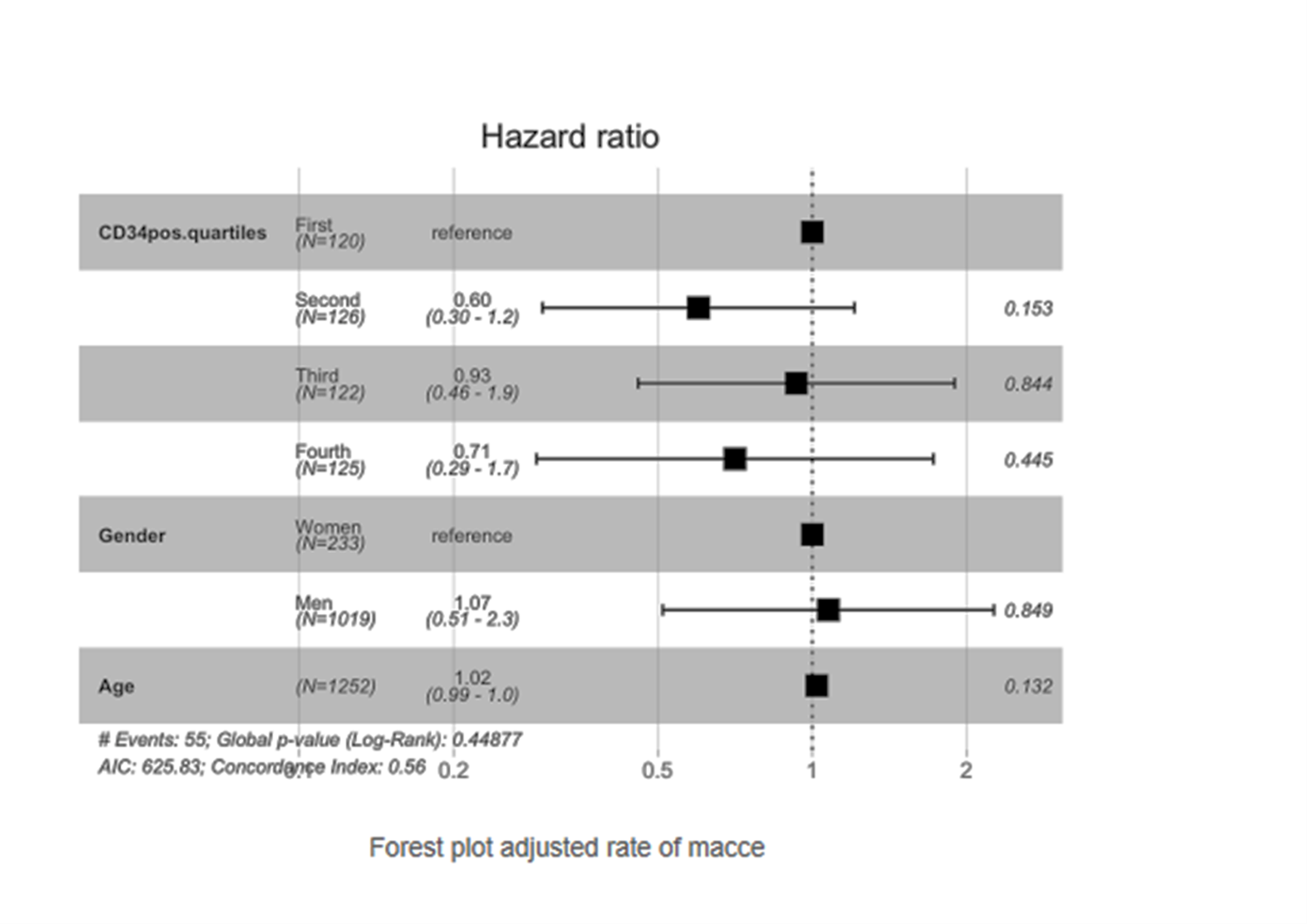


**Supplementary Figure 1** Forest plot with adjusted effect of bone marrow CD34+ cells stratified by quartiles, sex, and age on rate of major adverse cardiac and cerebrovascular event(s).
